# Supplementary material for: siRNA Features—Automated Machine Learning of 3D Molecular Fingerprints and Structures for Therapeutic Off-Target Data
Source: Int J Mol Sci. 2025 Jul 16;26(14):6795. doi: 10.3390/ijms26146795 (PMC12296190; doi:10.3390/ijms26146795)
Supplement: Supplementary file 1 [file ijms-26-06795-s001.zip › ijms-3589795-supplementary.pdf]

International Journal of
Molecular Sciences

MDPI

Article

Supplementary: siRNA Features – Automated Machine Learning of 3D Molecular Fingerprints and Structures for Therapeutic Off-Target Data

Michael Richter [1,*], Alem Admasu [2]

[1] Department of Chemistry, Binghamton University, Binghamton, NY 13902, USA
[2] Department of Physics and Astronomy, Rutgers University, Piscataway, NJ 08854, USA
* Correspondence: richter@binghamton.edu

S1: Dataset Description and Validation Set Configuration

Table 1 provides details for all datasets used. The size reflects the approximate number (≈30,000) of siRNA-gene interaction pairs analyzed after initial filtering (top 20% expressing genes based on RNA-Seq data). Descriptions aim for clarity and consistency with the main manuscript.

Table 1. Detailed description of datasets used in this study.

ID	Description	Features
0	Distances from MD trajectories; ECFP fingerprints for modifications	896
1	Full xyz coordinates from MD trajectories	2637
2	Simple numerical encoding (A/C/G/U/mods)	42
3	ECFP fingerprints (radius 1, nBits 32) per siRNA/mRNA position	1344
4	ECFP fingerprints (radius 2/1) for modifications + Gene Index	99
6N	Distances from minimized structures (base ref); RNA distances included	879
6R	As 6N but distances rescaled (0–255); Rigid AGO2 alignment	842
7N	Distances from minimized structures (4f3t ref); No RNA distances*	837
7R	As 7N but distances rescaled (0–255); Energy-minimized AGO2 align	800

*Train/Val split is 90%/10% & refers to the initial partitioning based on stratified sampling (See Section S4: Validation Set Configuration).

*RNA distances excluded due to incomplete residue information in the 4f3t reference structure for comprehensive calculation across all siRNAs.

Each dataset was preprocessed using standard normalization techniques appropriate for the feature type (e.g., z-score for numerical distances, one-hot for categorical). Feature selection methods like recursive feature elimination (RFE) were *not* performed for the results presented; rather, the performance of predefined feature sets (datasets) was compared directly.

For model training and initial validation partitioning, we used a stratified scheme to maintain the class distribution (approx. 18% positive cases). Specific validation strategies varied depending on the optimization phase, as detailed in Section S4. Cross-validation experiments employed stratified k-fold with k=10 for hyperparameter optimization (details in Section S10) and k=20 for metric optimization (details in Section S9).

All reported performance metrics refer exclusively to the validation set defined for each specific experimental phase. The validation strategy was chosen to suit the goal of each phase:

 https://doi.org/10.3390/ijms26146795

- *Hyperparameter Optimization (HPO - results shown in Main Paper Figure 4):* Utilized a predefined 10% stratified validation set derived from the initial 90/10 split. This set was passed to AutoGluon as `tuning_data` to guide hyperparameter selection. (Configuration details in Section S10).
- *Metric Optimization (results shown in Main Paper Figure 5):* Employed 20-fold stratified cross-validation (`n_splits=20`). Performance was evaluated on the internal validation folds generated by `StratifiedKFold`, allowing robust assessment across multiple data subsets. (Configuration details in Section S9).
- *Stack Level and Time Optimization (results shown in Main Paper Figure 6):* Utilized AutoGluon's internal holdout mechanism, automatically reserving 5% of the training data (`holdout_frac=0.05`) as a validation set for guiding model stacking and evaluating time-constrained performance. (Configuration details in Section S7 and S8).

S2: Supplementary Equations

Standard definitions for performance metrics used in this study:

$$\text{Accuracy} = \frac{\text{TP} + \text{TN}}{\text{TP} + \text{FP} + \text{FN} + \text{TN}} \tag{S1}$$

$$\text{Precision} = \frac{\text{TP}}{\text{TP} + \text{FP}} \tag{S2}$$

$$\text{Recall (TPR)} = \frac{\text{TP}}{\text{TP} + \text{FN}} \tag{S3}$$

$$\text{Specificity} = \frac{\text{TN}}{\text{TN} + \text{FP}} \tag{S4}$$

$$\text{F1-score} = \frac{2 \times \text{TP}}{2 \times \text{TP} + \text{FP} + \text{FN}} \tag{S5}$$

$$Q_{2,\text{rnd}} = \frac{(\text{TP} + \text{FN})(\text{TP} + \text{FP}) + (\text{TN} + \text{FP})(\text{TN} + \text{FN})}{(\text{TP} + \text{FP} + \text{FN} + \text{TN})^2} \tag{S6}$$

Note: $Q_{2,\text{rnd}}$ represents the expected accuracy of a random classifier based on class distributions. The denominator represents the square of the total number of instances. TP=True Positives, TN=True Negatives, FP=False Positives, FN=False Negatives.

S3: Hyperparameter Tuning Configuration (corresponding to Main Paper Figure 4)

For the hyperparameter optimization (HPO) results presented in Main Paper Figure 4, we used 10-fold stratified cross-validation (`n_splits=10`, `random_state=2222`) to generate initial training and validation splits (90%/10%). The AutoGluon configuration shown conceptually in Listing 1 was applied within each fold of this cross-validation. Accuracy (`eval_metric='accuracy'`) served as the optimization metric guiding the HPO process, and the 10% validation split derived from the outer fold was passed as `tuning_data` to AutoGluon's HPO routine.

```
# Requires pandas (pd), sklearn.model_selection (StratifiedKFold), autogluon.
    tabular (TabularPredictor)
# Assumes X, y (full dataset features and labels) are defined
# Setup 10-fold StratifiedKFold
skf = StratifiedKFold(n_splits=10, shuffle=True, random_state=2222)

# Loop iterating through the 10 folds generated by skf.split(X, y)
# for train_index, val_index in skf.split(X, y):
#     # Split data for this fold
#     X_train, X_val = X.iloc[train_index], X.iloc[val_index]
#     y_train, y_val = y.iloc[train_index], y.iloc[val_index]

    # --- AutoGluon Fit Call (Representative for one fold) ---
    # Instantiate predictor for this fold
    predictor = TabularPredictor(
        label='log2FC',                # Target variable column name
        problem_type='binary',         # Classification task
        eval_metric='accuracy',        # Metric guiding HPO and internal model
     selection
        path=f'AutogluonModels_HPO/fold_{fold_index}/' # Example save path
    per fold
    )
    # Fit predictor, performing HPO
    predictor.fit(
        train_data=pd.concat([X_train, y_train], axis=1), # Training data
    (90% of original) for this fold
        tuning_data=pd.concat([X_val, y_val], axis=1),   # Validation data
    (10% of original) for HPO guidance
        presets='best_quality',                          # Use high-quality
    base models
        time_limit=10862,                                # Example overall
    time limit per fold from source
        hyperparameters=hyperparameters,                 # Defined search
    spaces (See Section S11)
        hyperparameter_tune_kwargs={                     # HPO specific
    settings
                'num_trials': 2,                         # Number of HPO
    trials per base model type
                'scheduler': 'local',                    # HPO scheduler
                'searcher': 'random',                    # HPO search
    strategy
                'num_cpus': 64,                          # Parallel resources
     for HPO
        },
        use_bag_holdout=True, # Enable bagging; HPO uses tuning_data for
    model selection/stopping within HPO trials
        # Bagging parameters like num_bag_folds / num_bag_sets apply after
    HPO to potentially ensemble models.
    )
# --- End of outer loop ---
# Final performance metrics (as shown in Main Paper Figure 4) are typically
    aggregated across folds.
```

Listing 1: AutoGluon setup concept for hyperparameter optimization (corresponding to Main Paper Figure 4).

S4: Complete Confusion Matrix Elements

Dataset 0 consistently yielded non-predictive models (metrics often zero) in initial AutoGluon runs (as noted in main paper Table 2 discussion) and was therefore excluded from the detailed confusion matrix analysis presented here. Table 2 shows the confusion matrix elements (TP, FP, TN, FN) for the best-performing models identified during the

metric optimization phase (corresponding to performance data presented in Main Paper Figure 5).

Table 2. Complete confusion matrix elements for best-performing models from metric optimization (corresponding to Main Paper Figure 5 runs).

Dataset	TP	FP	TN	FN	Seed
Dataset 2	372	145	2175	277	2229
Dataset 3	370	146	2174	279	2229
Dataset 4	367	159	2161	282	2229
Dataset 6R	287	144	2176	362	2231
Dataset 7N	288	155	2165	361	2231
Dataset 7R	355	196	2123	294	2231

Metrics computed on internal validation folds of 20-fold CV.

S5: Hyperparameter Search Spaces

The following hyperparameter search spaces were used for the gradient boosting machine (GBM), XGBoost (XGB), and k-nearest neighbors (KNN) models during the hyperparameter optimization (HPO) phase detailed in Section S10. These definitions utilize AutoGluon/Ray Tune syntax (requiring imports like 'Int', 'Real', 'Categorical' from 'autogluon.core.space' or 'ray.tune.space') and 'numpy' for the KNN Mahalanobis check.

S11.1: GBM Hyperparameters

```
# Requires: from autogluon.core.space import Int, Real, Categorical
hyperparameters = {
    # 'GBM' typically maps to LightGBM in AutoGluon presets
    'GBM': [
        {
            'num_leaves': Int(10, 200, default=31),           # Max leaves in
    one tree
            'learning_rate': Real(0.01, 0.2, default=0.05), # Step size
    shrinkage
            'feature_fraction': Real(0.5, 1.0, default=0.9),# Feature
    subsampling (like colsample_bytree)
            'min_data_in_leaf': Int(20, 100, default=20),   # Minimum samples
     per leaf
            'extra_trees': Categorical(True, False),        # Whether to use
    extremely randomized trees logic
            'lambda_l1': Real(1e-8, 10.0, default=1e-3),    # L1
    regularization term
            'lambda_l2': Real(1e-8, 10.0, default=1e-3),    # L2
    regularization term
        } for i in range(3)  # Explore 3 different GBM configurations
    randomly drawn from this space per HPO trial run
    ]
}
```

Listing 2: Hyperparameter search space for LightGBM (GBM) used in HPO.

S11.2: XGBoost Hyperparameters

```
# Requires: from autogluon.core.space import Int, Real
hyperparameters = {
    'XGB': [
        {
            'n_estimators': Int(50, 300, default=100),      # Number of
    boosting rounds
```

```
            'eta': Real(0.01, 0.3, default=0.05),          # Learning rate (
    alias: learning_rate)
            'gamma': Real(0, 5, default=0),                # Minimum loss
    reduction required to make a split (alias: min_split_loss)
            'max_depth': Int(3, 9, default=6),             # Maximum depth of
    a tree
            'min_child_weight': Int(1, 10, default=1),     # Minimum sum of
    instance weight (hessian) needed in a child
            'subsample': Real(0.5, 1, default=0.8),        # Fraction of
    training instances sampled per tree
            'colsample_bytree': Real(0.5, 1, default=0.8), # Fraction of
    columns (features) sampled per tree
            'colsample_bylevel': Real(0.5, 1, default=0.8),# Fraction of
    columns sampled per split level
            'lambda': Real(1e-3, 10, log=True, default=1),# L2 regularization
     term (alias: reg_lambda)
            'alpha': Real(1e-3, 10, log=True, default=0), # L1 regularization
     term (alias: reg_alpha)
        } for i in range(5)  # Explore 5 different XGB configurations
    randomly drawn from this space per HPO trial run
    ]
}
```

Listing 3: Hyperparameter search space for XGBoost used in HPO.

S11.3: KNN Hyperparameters

```
# Requires: from autogluon.core.space import Int, Categorical
# Requires: import numpy as np
# Assumes X_train (training features DataFrame/array for the current fold) is
     defined in scope for Mahalanobis check

hyperparameters = {
    'KNN': [
        {
            'weights': Categorical('uniform', 'distance'), # Weighting
    function for neighbors
            'n_neighbors': Int(1, 50, default=5),          # Number of
    neighbors (k)
            'leaf_size': Int(10, 100, default=30),         # Leaf size passed
    to BallTree or KDTree
            'algorithm': Categorical('auto', 'ball_tree', 'kd_tree', 'brute')
    , # Algorithm used to compute nearest neighbors
            'metric': Categorical('minkowski', 'euclidean', 'manhattan',
                                  'chebyshev', 'mahalanobis'), # Distance
    metric
            'n_jobs': Categorical(-1, 1, 2, 4, 8),            # Number of
    parallel jobs (-1 uses all CPUs)
            'metric_params': Categorical(                     # Additional
    keyword arguments for the distance metric function
                None,  # Default for most metrics (e.g., Euclidean, Minkowski
    )
                # Conditionally define 'V' (inverse covariance matrix) ONLY
    if metric is 'mahalanobis'
                # AND the training data allows for stable calculation of the
    inverse covariance.
                *([{'V': np.linalg.inv(np.cov(X_train, rowvar=False))}]
                  # Check 1: Is the selected metric 'mahalanobis'? (Implicit
    if this branch is chosen)
                  # Check 2: Does training data (X_train) exist and have 2
    dimensions?
                  if hasattr(X_train, 'shape') and len(X_train.shape) == 2
```

```
                    # Check 3: Are there more samples than features? (
    Necessary for non-singular covariance)
                    and X_train.shape[0] > X_train.shape[1]
                    # Check 4: Is the covariance matrix invertible (non-
    singular)?
                    # Use try-except block for robustness against potential
    numerical issues during determinant calculation.
                    and (lambda cov_matrix: np.linalg.det(cov_matrix) != 0)(
    np.cov(X_train, rowvar=False))
                 # If any check fails, provide an empty list, effectively
    passing no extra args (i.e., 'metric_params': None)
                 else [])
          ),
      } for _ in range(10) # Explore 10 different KNN configurations
    randomly drawn from this space per HPO trial run
    ]
}
```

Listing 4: Hyperparameter search space for KNN used in HPO.

S6: Cross-Validation Performance (Metric Optimization - corresponding to Main Paper Figure 5)

For the results presented in Main Paper Figure 5 (and associated Tables 2, 4, 3), we employed 20-fold stratified cross-validation (`n_splits=20`) to robustly evaluate performance across various metrics. Key configuration aspects are shown conceptually in Listing 5. Table 3 summarizes the *best value achieved for each individual metric* across all seeds tested during this phase; note that the seed yielding the best value for one metric may differ from the seed yielding the best value for another metric or the best overall model performance.

```
# Assumes imports: from sklearn.model_selection import StratifiedKFold
# Assumes CFG is an object holding configuration, including CFG.seed (e.g.,
    2229)
n_splits = 20
# CFG.seed is set for each run iteration.
skf = StratifiedKFold(n_splits=n_splits, shuffle=True, random_state=CFG.seed)

# Inside the loop over folds generated by skf.split(X, y):
#   For each fold:
#     An AutoGluon TabularPredictor is instantiated and fit on the training
    portion of the fold.
#     Key AutoGluon parameters used within the loop for this phase include:
#       presets='best_quality' # Or similar high-quality preset
#       use_bag_holdout=True   # Critical for using AutoGluon with external
    CV loop
#       time_limit=600         # Example time limit per fold
#       # eval_metric could be set to one of the 25 tested metrics or a
    default (e.g., accuracy)
#       # No tuning_data is passed; validation performance is implicitly
    evaluated on the holdout part of the fold managed by StratifiedKFold.
#   Metrics are computed on the predictions made on the validation part of
    each fold and aggregated.
```

Listing 5: StratifiedKFold configuration concept for metric optimization (corresponding to Main Paper Figure 5).

Table 3. Best performance metrics achieved during metric optimization (results corresponding to Main Paper Figure 5 runs).

Dataset	Metric	Best Value	Seed
Dataset 2	Accuracy	0.7764	2229
Dataset 2	Precision	0.7198	2229
Dataset 2	Recall (TPR)	0.5731	2226
Dataset 2	Specificity	0.9384	2229
Dataset 2	F1-Score	0.5828	2229
Dataset 3	Accuracy	0.7774	2229
Dataset 3	Precision	0.7175	2229
Dataset 3	Recall (TPR)	0.5697	2225
Dataset 3	Specificity	0.9358	2229
Dataset 3	F1-Score	0.5849	2229
Dataset 4	Accuracy	0.7794	2229
Dataset 4	Precision	0.6983	2223
Dataset 4	Recall (TPR)	0.5650	2229
Dataset 4	Specificity	0.9348	2223
Dataset 4	F1-Score	0.5895	2229
Dataset 6R	Accuracy	0.7548	2231
Dataset 6R	Precision	0.6659	2223
Dataset 6R	Recall (TPR)	0.4415	2222
Dataset 6R	Specificity	0.9458	2227
Dataset 6R	F1-Score	0.4715	2222
Dataset 7N	Accuracy	0.7460	2231
Dataset 7N	Precision	0.6498	2224
Dataset 7N	Recall (TPR)	0.4434	2222
Dataset 7N	Specificity	0.9320	2224
Dataset 7N	F1-Score	0.4687	2225
Dataset 7R	Accuracy	0.7548	2231
Dataset 7R	Precision	0.6442	2230
Dataset 7R	Recall (TPR)	0.5472	2230
Dataset 7R	Specificity	0.9245	2231
Dataset 7R	F1-Score	0.5653	2230

Reports the best value achieved for each specific metric across all tested seeds during the metric optimization phase (detailed in Section S9). Note: The seed yielding the best value for one metric may differ from the seed yielding the best overall model performance.
All metrics reported are computed exclusively on the internal validation folds generated by the 20-fold CV used in the metric optimization phase.

S7: Random vs. Actual Accuracy Comparison

$Q_{2,\text{rnd}}$ (defined in Section S1, Equation S6) represents the expected accuracy of a random classifier based only on the dataset's class frequencies. The difference between a model's actual accuracy and $Q_{2,\text{rnd}}$ indicates the predictive improvement over random chance. Table 4 compares these values for the best models identified during the *metric optimization* phase (corresponding to Main Paper Figure 5 runs).

Table 4. Comparison of actual accuracy vs. random accuracy ($Q_{2,\mathrm{rnd}}$) from metric optimization (corresponding to Main Paper Figure 5 runs).

Dataset	Accuracy	Random Accuracy ($Q_{2,\mathrm{rnd}}$)	Difference	Seed
Dataset 2	0.7764	0.6528	0.1236	2229
Dataset 3	0.7774	0.6532	0.1242	2229
Dataset 4	0.7794	0.6514	0.1280	2229
Dataset 6R	0.7548	0.6447	0.1101	2231
Dataset 7N	0.7460	0.6441	0.1019	2231
Dataset 7R	0.7548	0.6462	0.1086	2231

All metrics reported in this table are computed exclusively on the internal validation folds generated during the 20-fold CV metric optimization runs.

S8: Stack Level and Time Optimization (corresponding to Main Paper Figure 6)

This phase utilized AutoGluon's `roc_auc_ovo_macro` as the internal evaluation metric for guiding model selection and stacking within time limits. Final performance, reported in Table 5, uses PRC AUC as it is more suitable for evaluating performance on this imbalanced dataset. The highest PRC AUC achieved for Dataset 3 was 0.785 (Seed 2162, Stack Level 6), slightly higher than the value shown in the main paper figure due to specific run variations or rounding.

Table 5. Optimization of stack level and computation time across datasets (results corresponding to Main Paper Figure 6).

Dataset ID	Seed	PRC AUC	Time (s)	Stack Level
3	2162	0.785	370	6
3	5836	0.783	355	6
3	5836	0.784	295	5
7R	4389	0.736	780	4
7N	7414	0.729	980	4

All metrics reported are computed exclusively on the 5% validation holdout set generated internally by AutoGluon during the stack level optimization runs.

S9: Model Training Configuration (Stack Level Optimization)

Listing 6 shows the key AutoGluon TabularPredictor configuration used for the stack level and time optimization runs detailed in Section S7 (corresponding to Main Paper Figure 6).

```
# Assumes imports: from autogluon.tabular import TabularPredictor
# Assumes train: training data (Pandas DataFrame)
# Assumes time_limit: integer, seconds (e.g., 370, 355, etc.)
# Assumes seed: random seed (integer, e.g., 2162, 5836, etc.)
predictor = TabularPredictor(
    label='log2FC',                    # Target variable column name
    problem_type='binary',             # Classification type
    eval_metric='roc_auc_ovo_macro', # Internal optimization metric for stack
    /time opt
    path=f'AutogluonModels_Stack/'  # Example directory to save models
).fit(
    train_data=train,
    presets='best_quality',            # Use high-quality model configurations
    time_limit=time_limit,             # Maximum training time in seconds
    holdout_frac=0.05,                 # Reserve 5% of training data for
    internal validation
    num_bag_folds=2,                   # Number of folds for bagged ensembles (
    example value)
```

```
    num_bag_sets=1,                 # Number of bagging iterations (example
    value)
    ag_args_fit={'num_cpus': 64, 'seed': seed} # Arguments for individual
    model fits
)
```

Listing 6: AutoGluon TabularPredictor configuration for stack level optimization (corresponding to Main Paper Figure 6).

For consistent model comparison, we designated Precision-Recall Area Under the Curve (PRC AUC) as our primary evaluation metric, particularly for Dataset 3 which demonstrated the best overall performance. This metric was selected because it better handles imbalanced datasets by focusing on the minority class performance, which is critical for off-target prediction tasks where false positives can have significant practical consequences. While we report multiple metrics (defined in Section S1) to provide a comprehensive assessment of model performance, conclusions regarding the superiority of different feature engineering approaches are based primarily on PRC AUC values.

Supplementary Data Files and Code

Code for feature generation, model training using AutoGluon, and evaluation scripts developed for this study are publicly available at https://github.com/mrichter0/siRNA-Features. Processed datasets used for training and validation, containing the final feature vectors and target labels, are also now available in the repository. These files enable full reproduction of the key modeling results presented. Raw RNA-Seq data are subject to size and potential access constraints and are also available upon reasonable request.
